# A Novel Formulation of Cisplatin with γ-Polyglutamic Acid and Chitosan Reduces Its Adverse Renal Effects: An In Vitro and In Vivo Animal Study

**DOI:** 10.3390/polym13111803

**Published:** 2021-05-30

**Authors:** Masao Sasai, Kazuma Sakura, Takayuki Matsuda, Hiroshi Uyama

**Affiliations:** 1 Division of Translational Research, Osaka University Hospital, Suita 565-0871, Japan; sasai-masao@tissue.med.osaka-u.ac.jp; 2 Respiratory Center, Osaka University Hospital, Suita 565-0871, Japan; 3 Department of Surgery, Graduate School of Medicine, Osaka University, Suita 565-0871, Japan; 4 Department of Applied Chemistry, Graduate School of Engineering, Osaka University, Suita 565-0871, Japan; t.matsuda.OU2011@gmail.com (T.M.); uyama@chem.eng.osaka-u.ac.jp (H.U.)

**Keywords:** malignant pleural mesothelioma, *cis*-diamminedichloroplatinum (II), CDDP, controlled release, adverse events, chemotherapy, antitumor efficacy, γ-polyglutamic acid, chitosan, cisplatin

## Abstract

Cisplatin (*cis*-diamminedichloroplatinum (II); CDDP) is a key chemotherapeutic agent but causes renal damage and other off-target effects. Here, we describe the pharmacological and biochemical characteristics of a novel formulation of CDDP complexed with γ-polyglutamic acid (γ-PGA) and chitosan (CS), γ-PGA/CDDP-CS, developed by complexing CDDP with γ-PGA, then adding CS (15 kDa; 10 mol%/γ-PGA). We analyzed tumor cytotoxicity in vitro, as well as blood kinetics, acute toxicity, and antitumor efficacy in vivo in BALB/cAJcl mice. γ-PGA/CDDP-CS showed pH-dependent release in vitro over 12 days (9.1% CDDP released at pH 7.4; 49.9% at pH 5.5). It showed in vitro cytotoxicity in a dose-dependent manner similar to that of uncomplexed CDDP. In a mesothelioma-bearing mouse model, a 15 mg/kg dose of CDDP inhibited tumor growth regardless of the type of formulation, complexed or uncomplexed; however, all mice in the uncomplexed CDDP group died within 13 days. γ-PGA/CDDP-CS was as effective as free CDDP in vivo but much less toxic.

## 1. Introduction

Cisplatin (*cis*-diamminedichloroplatinum (II); CDDP) is an important antitumor agent indicated for many solid cancers. However, its clinical application is limited by serious adverse effects. Renal damage, one of its adverse reactions, can cause acute renal failure if untreated. Another variant of platinum therapy, carboplatin, was developed to reduce off-target damage, but renal function remains at risk and requires close monitoring; therefore, its usability is limited [1,2]. Formulations that regulate drug release over time generally reduce its off-target effects and allow for the use of higher doses. For example, micellization of CDDP using polyethylene glycol (PEG) has been reported as a method for reducing the nephrotoxicity of CDDP while exerting an equivalent antitumor effect [3]. However, owing to safety concerns associated with the use of PEG, it is not widely used clinically. Furthermore, different adverse events have been reported with PEGylated liposomal doxorubicin (Doxil) formulations compared with that with uncomplexed doxorubicin formulations. Therefore, a new formulation that reduces the adverse effects that may be caused by PEGylation, such as those observed with Doxil, will be useful.

Malignant pleural mesothelioma is an intractable tumor related to asbestos exposure; its associated morbidity is a social concern, considering the use of asbestos in Eastern Europe, China, and India. Current treatments involve chemotherapy, surgery, and radiation. Although the use of immunotherapy for mesothelioma is improving, platinum drug chemotherapy is considered the first-line treatment [4]. 

γ-Polyglutamic acid (γ-PGA), a common glutamic acid polymer, is water-soluble, anionic, biodegradable, non-immunogenic, and edible; thus, it has been utilized effectively in many pharmaceutical applications [5,6,7,8,9,10,11,12]. A previous report showed that a γ-PGA-CDDP conjugate reduced the toxic effects of CDDP and also demonstrated antitumor activity [6]. However, it is feared that the in vivo stability of such conjugate formulations is weaker than that of particles. Another paper reported the creation of a sustained-release drug formulation by using zwitterionic polymers as drug delivery carriers [13,14]. However, the biodistribution of such formulations was reported to be mostly in the kidneys, which was considered unsuitable for CDDP formulations. Chitosan (CS) is a deacetylated derivative of chitin, a polysaccharide derived from the hard exoskeleton of shellfish. When applied in medicine, it alters drug solubility to allow controlled release of drugs into the system; this effect is, in part, due to the cationic character imparted by its primary amino groups [15,16]. Chitosan-based nanoparticles are emerging as one of the most promising delivery vehicles for cancer chemotherapy and diagnosis because of their unique characteristics, such as biodegradability, biocompatibility, cell membrane penetrability, high drug-carrying capacities, pH-dependent therapeutic unloading, multi-functionality, and prolonged circulating time [17,18,19,20,21,22]. 

**Figure 1:**
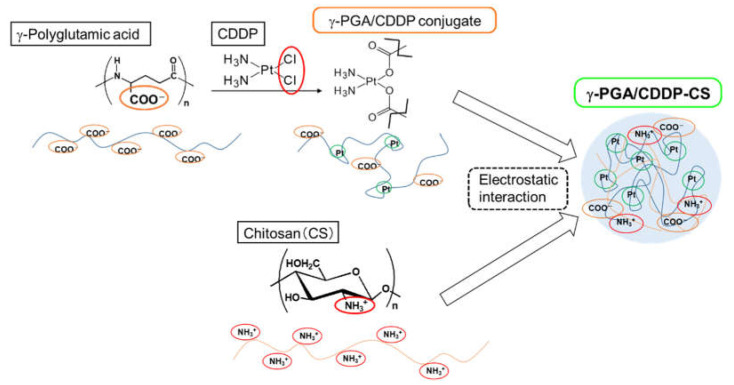
Schema of synthesis of γ-PGA/CDDP-CS.

To improve CDDP therapy, we developed a novel drug formulation by complexing CDDP with γ-PGA and CS, based on the hypothesis that the pharmacokinetic-modifying characteristics of γ-PGA and CS would improve the safety and efficacy profiles of CDDP (Figure 1).

First, γ-polyglutamic acid (γ-PGA) and cisplatin (cis-diamminedichloroplatinum (II); CDDP) were covalently bonded to form the γ-PGA/CDDP conjugate. Second, the γ-PGA/CDDP conjugate and chitosan (CS) were mixed to form γ-PGA/CDDP-CS particles via electrostatic interactions. 

## 2. Materials and Methods

### 2.1. Preparation of γ-PGA/CDDP-CS

We purchased γ-PGA from BioLeaders Corporation (Yongin, Korea), CS from Amicogen Inc. (Jinju, Korea), and CDDP from Tokyo Chemical Industry Co. (Tokyo, Japan). CDDP was stirred and dissolved in a saline solution at a concentration of 20 or 40 mol%. Next, 75 mg of γ-PGA (2000 kDa) was added and the solution was stirred continuously at 37 °C for 48 h. Finally, the γ-PGA/CDDP conjugate solution was dialyzed (molecular weight cutoff: 2000 Da) and lyophilized. 

We reconstituted 10 mL of the γ-PGA/CDDP conjugate solution at 1 mg/mL and adjusted its pH to 9.0 using 0.1 M NaOH. CS (15 kDa) was added dropwise (1.25, 2.5, and 5 mL) to produce one of the three final concentrations at 1.0 mL/h using a micro-syringe pump (KDS100, KD Scientific, Hollistor, MA, USA). The final γ-PGA/CDDP-CS solution was filtered through a 1.2 µm filter. The particle size was determined using a DLS-6006 US spectrophotometer (Otsuka Electronics Co., Osaka, Japan). 

### 2.2. Measurement of CDDP Release In Vitro

Three milliliters of γ-PGA/CDDP-CS solution (300 µg/mL) was placed into a dialysis membrane (MWCO: 2000 Da) and the membrane was immersed in 60 mL of one of the two compositions of phosphate-buffered saline (PBS) (pH 7.4 and pH 5.5, 70 mM NaCl). Three microliters of the external PBS solution was extracted at each time point (1, 2, 3, 6, and 12 h and 1, 2, 3, 5, and 7 days), and the CDDP concentration was measured by inductively coupled plasma (ICP) emission analysis (Spectrum One System B2, Perkin Elmer, MA, USA). 

### 2.3. In Vitro Cytotoxicity

A murine cell line (AB22) was provided by Dr. Cleo Robinson and Dr. Bruce Robinson of the University of Western Australia [23]. After 2 cycles of in vivo selection, AB23G2 murine mesothelioma cells were isolated from the AB22 cell line and cultured for use in the present study. Cytotoxicity against AB23G2 tumor cells was measured using the 3-(4,5-di-methylthiazol-2-yl)-2,5-diphenyltetrazolium bromide yellow tetrazole (MTT) assay. Briefly, a total of 3000 AB23G2 cells/well were seeded into a 96-well plate. The test compound (50 µL of CDDP-saline, γ-PGA/CDDP-CS, γ-PGA-CS, or saline) was added, and the assay was performed on the samples on Days 1, 3, 5, and 7 after seeding. γ-PGA/CDDP-CS was tested at concentrations of 0, 10, 50, and 100 μg/mL.

### 2.4. Biodistribution of γ-PGA/CDDP-CS in Normal Mice

Female BALB/cAJcl mice (6–8 weeks old) were obtained from CLEA Japan (Tokyo, Japan) and kept in standard housing with food and water ad libitum. The animals used in all experiments were provided with an acclimatization period of at least 1 week in the breeding room of the Animal Experimentation of Osaka University Graduate School of Medicine. The *in vivo* experiments were performed using a protocol approved by the Ethics Review Committee for Animal Experimentation of Osaka University Graduate School of Medicine (#21-055-0). Test compounds and vehicle controls were administered intraperitoneally (ip). Blood CDDP concentrations were measured 1, 2, 24, 72, and 168 h after administration by ICP emission analysis. These samples were obtained by incineration of the collected blood samples. 

### 2.5. In Vivo Acute Toxicity

CDDP (7.5 or 15 mg/kg), γ-PGA/CDDP-CS (7.5 or 15 mg/kg), and saline (vehicle control) were injected ip into AB23G2-bearing mice, and they were assessed up to 11 days after administration (*n* = 5 each). Survival times were recorded to construct Kaplan–Meier survival curves. Further, to assess acute nephrotoxicity, CDDP (*n* = 4) and γ-PGA/CDDP-CS (*n* = 4) were administered (ip) at doses of 4.5 mg/kg and 45 mg/kg, respectively. Histological changes due to tubular necrosis were quantitated by determining the percentage of tubules with evident cell necrosis and tubule dilatation, as follows: 0 = none, 1 = ≤10%, 2 = 11–25%, 3 = 26–45%, and 4 = ≥46% [24].

### 2.6. Antitumor Efficacy of γ-PGA/CDDP-CS in a Subcutaneous Tumor-Bearing Mouse Model

AB23G2 cells (4.8 × 10^6^ cells/mouse) were subcutaneously inoculated into the right dorsolateral region of mice. After 9 days of cell inoculation, nodule diameters were measured, mice were divided into groups such that each group had a similar tumor size, and the test compound (saline control (*n* = 5), CDDP (*n* = 5) or γ-PGA/CDDP-CS (*n* = 5)) was injected (ip). Subsequently, nodule diameters were measured on Days 12, 13, 14, 15, 20, and 22 after cell inoculation. The measurements were performed without anesthesia and in turns for each group.

### 2.7. Statistical Analysis

Student’s *t*-test was performed to determine statistical significance. All results were expressed as the mean ± standard error of the mean (SEM). Differences between the groups in the survival experiment were determined using the Kaplan–Meier log-rank test. In evaluations of histological changes, two groups were compared using the Mann–Whitney test. All values were considered statistically significant at a *p*-value < 0.05. 

## 3. Results

### 3.1. Characteristics of γ-PGA/CDDP-CS

**Figure 2:**
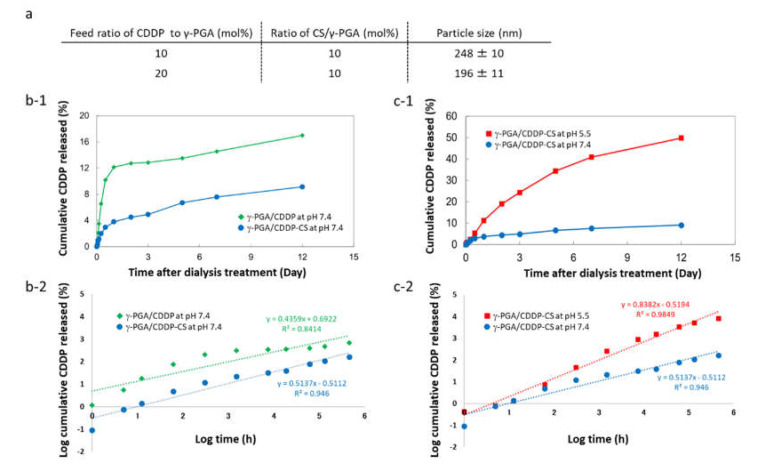
Characteristics of γ-PGA/CDDP-CS. (**a**) Particle size of γ-PGA/CDDP-CS. Data are represented as mean values ± standard error of the mean (SEM). (**b-1**) Cumulative CDDP released from γ-PGA/CDDP and γ-PGA/CDDP-CS 12 days after dialysis at pH 7.4 was 17.0% and 9.1%, respectively; (**b-2**) Korsmeyer–Peppas model: The approximate expressions for γ-PGA/CDDP and γ-PGA/CDDP-CS were calculated as *y* = 0.4359*x* + 0.6922 (*R*^2^ = 0.84) and *y* = 0.5137*x* − 0.5112 (*R*^2^ = 0.95), respectively. (**c-1**) Cumulative CDDP released from γ-PGA/CDDP-CS 12 days after dialysis at pH 7.4 and pH 5.5 (70 mM NaCl) was 9.1% and 49.9%, respectively. (**c-2**) Korsmeyer–Peppas model: The approximate expression for γ-PGA/CDDP-CS at pH 5.5 was calculated as *y* = 0.8382*x* − 0.594 (*R*^2^ = 0.98). The data shown are for one representative experiment.

The particle size of γ-PGA/CDDP-CS was found to be 196 ± 11 nm at a 10 mol% ratio of CS/γ-PGA and a 20 mol% feed ratio of CDDP to γ-PGA. The particle size was dependent on the composition of each formulation. A decrease in the amount of CDDP increased the particle size (Figure 2a). Upon measuring CDDP release through the dialysis membrane (i.e., the CDDP release rate), the cumulative CDDP released from the γ-PGA/CDDP-CS formulation at pH 7.4 amounted to 6.7% on Day 5 and 9.1% on Day 12, while that from the γ-PGA/CDDP conjugate (i.e., without CS) was 13.5% on Day 5 and 17% on Day 12 (Figure 2b-1). However, under pH 5.5/70 mM NaCl conditions, cumulative CDDP released from the γ-PGA/CDDP-CS formulation amounted to 34.3% on Day 5, demonstrating the pH-dependent property of the formulation (Figure 2c-1).

### 3.2. In Vitro Cytotoxicity against Murine Mesothelioma Cells

**Figure 3:**
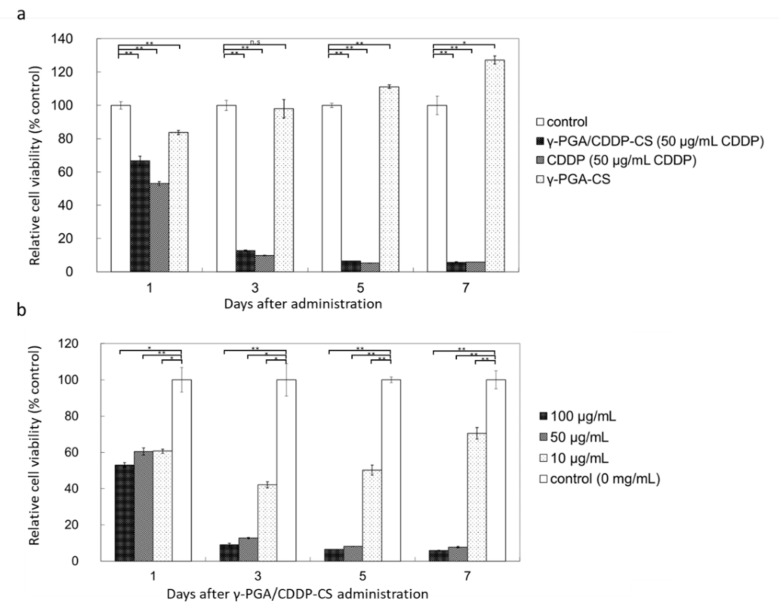
In vitro cytotoxicity of γ-PGA/CDDP-CS. (**a**) Relative cell viability compared with the control: time-dependent cytotoxicity was observed for all compounds (50 µg/mL CDDP), except γ-PGA-CS, which did not include CDDP. The values were 66.7% ± 2.7%, 53.0% ± 1.1%, and 83.7% ± 1.3% on Day 1; 12.7% ± 0.4%, 9.8% ± 0.2%, and 98.0% ± 5.6% on Day 3; 6.4% ± 0.1%, 4.7% ± 0.2%, and 111.2% ± 1.1% on Day 5; and 5.8% ± 3.4%, 5.0% ± 0.3%, and 127% ± 2.5% on Day 7 for γ-PGA/CDDP-CS, CDDP, and γ-PGA-CS, respectively. (**b**) Relative cell viability of γ-PGA/CDDP-CS compared with the control at a concentration of 10 μg/mL; cytotoxicity was slightly lower than that at 50 μg/mL and 100 μg/mL. The values for 100, 50, and 10 μg/mL of γ-PGA/CDDP-CS were 53.1% ± 1.2%, 60.4% ± 1.9%, and 60.7% ± 1.1% on Day 1; 9.1% ± 0.8%, 12.7% ± 0.4%, and 42.1% ± 1.7% on Day 3; 6.5% ± 0.0%, 8.1% ± 0.1%, and 50.2% ± 2.8% on Day 5; and 5.8% ± 0.2%, 7.7% ± 0.4%, and 70.4% ± 3.2% on Day 7, respectively. Data are represented as mean values ± standard error of the mean (SEM). Significant differences were analyzed by t-test (* *p* < 0.05, ** *p* < 0.01, n.s: Not significant).

The MTT assay showed that γ-PGA/CDDP-CS produced little cytotoxicity on Day 1, while the γ-PGA/CS conjugate alone produced no cytotoxicity (Figure 3a). 

No significant difference was observed between γ-PGA/CDDP-CS and free CDDP, when the trend of concentration-dependent cytotoxicity against murine mesothelioma cells was studied. γ-PGA/CDDP-CS cytotoxicity was concentration-dependent, and cell viability was 9.1% ± 0.8% at 100 µg/mL, 12.7% ± 0.4% at 50 µg/mL, and 42.1% ± 1.7% at 10 µg/mL compared with that of the control 3 days post-administration (Figure 3b). The 50% inhibitory concentration (IC_50_, µg/mL) was evaluated; it was 7.50, 9.26, and 12.5 for γ-PGA/CDDP-CS and 0.0063, 0.0018, and 0.0016 for free CDDP on Days 3, 5, and 7, respectively (free CDDP: data not shown).

### 3.3. In Vivo CDDP Concentration

**Figure 4:**
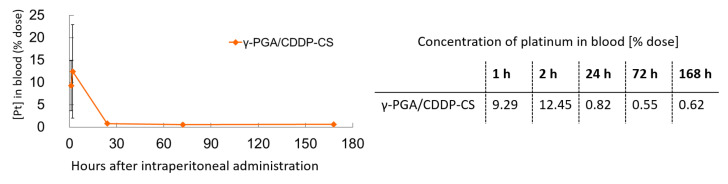
Blood concentration of γ-PGA/CDDP-CS in mice. γ-PGA/CDDP-CS was measured until 168 h after administration. The maximum concentration of platinum in the blood (% dose) was 12.5% ± 10.4%. Data are represented as mean values ± standard error of the mean (SEM).

CDDP in the blood was measurable 1 h after administration; it was detectable in the γ-PGA/CDDP-CS group for up to 168 h (0.62% of total CDDP injected) (Figure 4). 

### 3.4. Acute Toxicity of γ-PGA/CDDP-CS in Normal Mice

**Figure 5:**
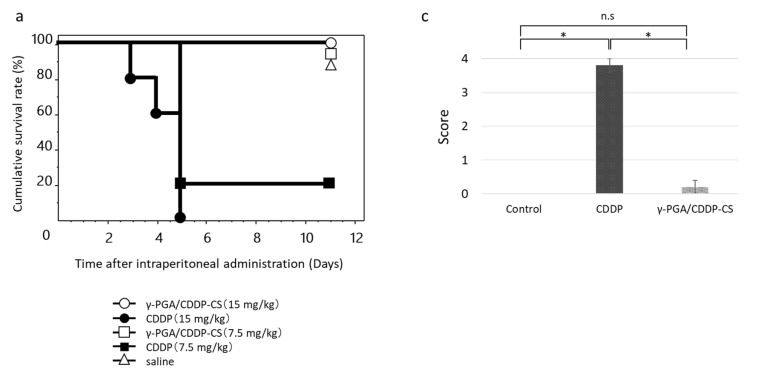

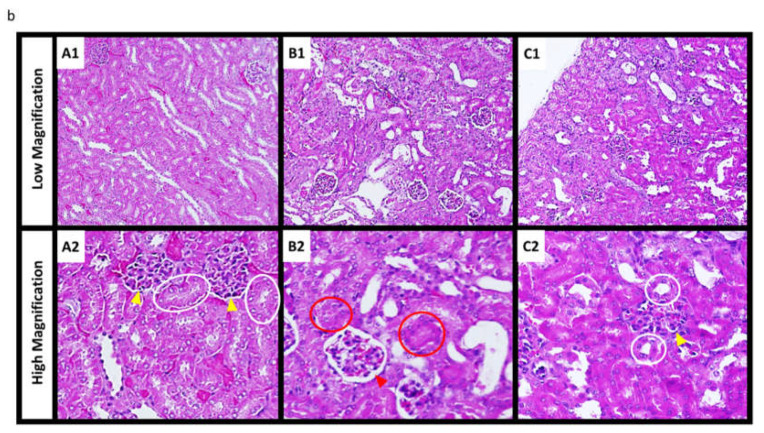
Acute toxicity after CDDP or γ-PGA/CDDP-CS administration. (**a**) The survival rate after CDDP (7.5, 15 mg/kg) and γ-PGA/CDDP-CS (7.5, 15 mg/kg) administration. The symbols represent the following: CDDP 15 mg/kg group (●), CDDP 7.5 mg/kg group (■), and γ-PGA/CDDP-CS group (15 mg/kg [◯], 7.5 mg/kg [□]). Differences between the groups in the survival experiment were determined using the Kaplan–Meier log-rank test. The mean survival time of mice that received treatment with each γ-PGA/CDDP-CS was significantly prolonged compared with that of mice treated with each free CDDP. Respectively, the differences between γ-PGA/CDDP-CS vs. CDDP, 15 vs. 7.5, 7.5 vs. 7.5, 15 vs. 15, and 7.5 vs. 15 mg/kg were considered statistically significant at *p* < 0.05, *p* < 0.05, *p* < 0.005, and *p* < 0.005. (**b**) The hematoxylin and eosin stained image of murine kidney shows no damage or damage to the proximal renal tubule in the saline group (A1: Low magnification; A2: High magnification) and the CDDP (4.5 mg/kg) group (B1: Low magnification; B2: High magnification). The white and red circles show a normal and damaged proximal renal tubule, respectively, while the yellow and red arrowheads show a normal and damaged Bowman’s capsule, respectively. No injury was observed in the control and γ-PGA/CDDP-CS (45 mg/kg) groups (C1: Low magnification; C2: High magnification). (**c**) Histological changes due to tubular necrosis were quantitated by determining the percentage of tubules with evident cell necrosis and tubule dilatation. Data are represented as mean values ± standard error of the mean (SEM). Statistical analysis was performed using the Mann–Whitney test, with the significance level at a *p*-value of <0.05 (*).

In the high-dose (15 mg/kg) CDDP-saline group, individual mice began to die on the third day after administration, and none (0%) survived beyond Day 5. In the low-dose (7.5 mg/kg) group, survival on Day 5 was 20%. In the high-dose (15 mg/kg) γ-PGA/CDDP-CS group, survival was 100% on Day 5 (Figure 5a). The mean survival time of mice that received treatment with CDDP or γ-PGA/CDDP-CS was significantly prolonged (*p* < 0.05) compared with that of mice treated with free CDDP. Histological analysis of the kidneys after administration of saline, 4.5 mg/kg CDDP, and 45 mg/kg γ-PGA/CDDP-CS revealed damage to the proximal renal tubule in the CDDP-saline group but not in the vehicle control or γ-PGA/CDDP-CS groups. Mesenchymal kidney tissue in the γ-PGA/CDDP-CS group was slightly edematous, but the renal tubules were intact (Figure 5b).

### 3.5. Antitumor Efficacy in a Murine Malignant Pleural Mesothelioma Model

**Figure 6:**
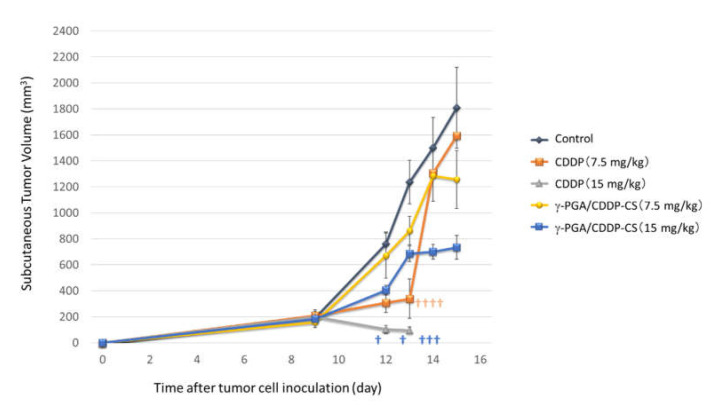
In vivo antitumor effect. AB23G2 cells were inoculated on the backs of mice. Each compound was administered 9 days after inoculation. In the CDDP high-dose (15 mg/kg) group (▲), all mice died within 14 days of inoculation and 5 days after administration. In the CDDP low-dose (7.5 mg/kg) group (■), 4 out of 5 died in 14 days. In the γ-PGA/CDDP-CS group (high-dose [■], low-dose [●]), tumor growth was suppressed on Days 13, 14, and 15. In the control group (◆), consistent tumor growth was observed. The † in the figure indicates that the mouse died. Data are represented as mean values ± standard error of the mean (SEM).

All mice in the high-dose and 80% in the low-dose CDDP groups had died by Day 14 after tumor cell implantation (Day 5 after drug administration). In the control group, the nodule diameter increased over time. In the high-dose γ-PGA/CDDP-CS group, a transient increase in the nodule diameter was suppressed in a dose-dependent manner from Days 13 to 15. However, in the low-dose group, the nodule diameter was similar to that in the control group (Figure 6).

## 4. Discussion

We developed a novel formulation of CDDP complexed with γ-PGA and CS to improve the safety and efficacy of CDDP-based chemotherapy, particularly in terms of renal damage. Our results suggest that the developed γ-PGA/CDDP-CS formulation was significantly less toxic to mouse kidney at a dose that was efficacious against mouse mesothelioma cells and tumors in vivo. 

The biochemical characteristics of nanoparticles can vary widely because of differences in size and surface charge, and can particularly affect hemolysis, platelet activation, plasma recalcification time, and cell viability and internalization over time [21,25]. In fact, the particle size of our formulation varied widely with changes in the ratio of CS and γ-PGA. When the amount of CDDP added increased, the structure of the polymer became condensed; consequently, the maximum diameter of γ-PGA/CDDP-CS decreased. Additionally, the modification of CDDP using CS and γ-PGA is suitable for clinical use because both these polymers are biodegradable and non-toxic. The concentration of cumulative CDDP released from the γ-PGA/CDDP-CS formulation was quite different from that released from the γ-PGA/CDDP conjugate without CS (Figure 2). At pH 7.4, the amount of CDDP released decreased in the formulation with chitosan. This may be due to chitosan, which is positively electrified and forms a complex through an electrification interaction, resulting in a stable formulation and thus inhibiting the release of CDDP [17]. Further, the key stability parameter for the retention of CDDP is pH, considering the influence of electrostatic interactions shielding the ionic species [26,27]. Given that the formulation released CDDP optimally at pH 5.5 and that tumor conditions are frequently acidic [28], we believe our formulation would be suitable for therapeutic purposes. Alternatively, it is conceivable that CDDP was released due to particle degradation in late endosomes upon uptake into cells. Indeed, our γ-PGA/CDDP-CS formulation possessed the same in vitro cytotoxicity as free CDDP. This cytotoxicity was consistent with previously reported studies, except that the pH of the culture medium did not fluctuate and was devised to adequately assess cell uptake [13,14].

Another advantage of γ-PGA/CDDP-CS is its prolonged blood circulation time. Our biodistribution studies demonstrated that the γ-PGA/CDDP-CS formulation, upon ip administration, was rapidly transferred to the bloodstream and remained in circulation for at least 7 days (Figure 4). Its concentration was slightly higher and the blood circulation time was longer than that of free CDDP previously reported [29]. We considered this to be a unique feature of this formulation because no other zwitterionic polymer formulation reported to date has been shown to maintain blood levels for such a long period of time. It was considered that during the early phase after ip administration, CDDP detected in the bloodstream was not free but was gradually released from the reticuloendothelial system (RES), where the formulation was captured after ip administration. The in vivo acute toxicity results suggested that even the 45 mg/kg dose of γ-PGA/CDDP-CS was not toxic. This is supported by the survival curve analysis, which shows that the survival curve of our formulation was strongly shifted to the right (indicating a much better survival rate than free CDDP) (Figure 5a). Furthermore, histological evidence of severe renal tubule damage was found in the kidneys of free CDDP-administered mice. However, this damage was absent in the control and γ-PGA/CDDP-CS groups despite a high CDDP concentration in the blood, suggesting that our formulation was not toxic to the kidneys (Figure 5b,c). Moreover, it is suggested that in the bloodstream, the formulation is not in the form of free CDDP that induces renal damage. Intraperitoneal administration of γ-PGA/CDDP-CS to normal mice resulted in a decrease in blood CDDP levels within 24 h; however, in a preliminary study, CDDP levels were elevated in the liver 3 days after administration (data not shown). These data suggest that γ-PGA/CDDP-CS might be trapped in the liver early after administration.

Based on the particle size (approximately 200 nm in diameter) and charge (negative charge: ζ potential −59 mV) of γ-PGA/CDDP-CS, it was considered that the particles were not taken up by the liver parenchyma but were bound to scavenger receptors on macrophages present in the sinusoids of the liver and phagocytosed [30,31]. However, no significant hepatic damage due to γ-PGA/CDDP-CS was observed upon macroscopic examination (data not shown). Although γ-PGA/CDDP-CS is not phagocytosed in the liver immediately after administration, it is thought to be gradually trapped in the liver during circulation; however, some amount of it may remain in the blood and escape degradation in the liver. The blood concentration of CDDP may be due to the escape of γ-PGA/CDDP-CS from the liver and free CDDP being released from the degradation of γ-PGA/CDDP-CS. As the blood concentration of CDDP was 0.123, 0.083, and 0.093 mg/kg 24, 72, and 168 h after γ-PGA/CDDP-CS administration, respectively, little antitumor effect was expected. However, in tumor-bearing mice, γ-PGA/CDDP-CS which escaped capture in the liver leaked into the tumor cells while circulating in the blood and released CDDP in the low pH tumor environment; this was thought to exert an antitumor effect. This was suggested because the antitumor effect was observed on the fourth day after administration of γ-PGA/CDDP-CS in the study of tumor-bearing mice (in vivo study). We hypothesized that this formulation did not degrade in the RES but was retained there prior to its release into the bloodstream over time. However, because CDDP was detectable in the blood until Day 7, we speculate that much of the γ-PGA/CDDP-CS exited the RES and was recaptured, and thus was metabolized gradually. The γ-PGA/CDDP-CS released from the RES accumulated in the tumor tissue and was then released CDDP in situ in a pH-dependent manner [32]. Moreover, the antitumor effect was observed on Day 6 after nanoparticle administration in vivo (Figure 6). Therefore, sustaining the antitumor effect with a single administration can enable lengthening of the administration interval, thereby reducing patient burden.

## 5. Conclusions

In summary, after administration, some of the γ-PGA/CDDP-CS particles were delivered to the tumor tissues, where they exerted their antitumor effects by releasing CDDP over several days; however, most of them were trapped and degraded by the RES such as the liver and were gradually released into the bloodstream as free CDDP—this improved their safety profile without affecting their antitumor efficacy. In this study, the toxicity of CDDP released from the γ-PGA/CDDP-CS formulation was greatly reduced and the safety profile greatly improved. For a more effective antitumor effect, it is necessary to adjust the particle charge to neutral or add steric hindrance by PEG modification to avoid particles getting trapped by the RES. Our results suggest the potential usefulness of γ-PGA/CDDP-CS in clinical chemotherapy, although many developmental factors remain. Through use of time-dependent anticancer agents, the antitumor efficacy may be better than that achieved by using concentration-dependent anticancer agents, even though the formulation trapped in the RES gradually enters the bloodstream in the form of free drug, which, in turn, is expected to improve its safety profile.
